# Structural Model of Medication Errors in Clinical Nursing: A Structural Equation Model Based on the Ecological Systems Theory

**DOI:** 10.1155/jonm/1245208

**Published:** 2026-03-16

**Authors:** Junekyu Kim, Yeoungsuk Song

**Affiliations:** ^1^ Department of Nursing, Kyungil University, Gyeongsan, South Korea, kiu.ac.kr; ^2^ College of Nursing, Research Institute of Nursing Innovation, Kyungpook National University, Daegu, South Korea, knu.ac.kr

**Keywords:** communication, fatigue, medication error, nurses, patient safety, professional practice

## Abstract

**Background:**

Medication errors remain a critical patient safety issue. Although prior research has examined individual and environmental determinants, few studies have investigated their complex interrelationships within an integrated theoretical framework, particularly in clinical nursing practice.

**Aim:**

To examine the structural relationships among the nursing work environment, communication with health professionals, fatigue, and medication errors among clinical nurses, grounded in Bronfenbrenner’s ecological systems theory.

**Methods:**

A cross‐sectional study was conducted involving 230 clinical nurses from four tertiary hospitals in South Korea. Data were collected using validated self‐report questionnaires measuring the nursing work environment, communication with health professionals, fatigue, and medication errors. Structural equation modeling, supported by confirmatory factor analysis, was used to evaluate direct and indirect pathways and to assess overall model fit.

**Results:**

The proposed model demonstrated acceptable fit (*χ* ^2^/df = 2.98, RMSEA = 0.08, CFI = 0.93). The nursing work environment exerted a significant direct effect on communication (*β* = 0.80, *p* < 0.001), and communication was significantly associated with reduced fatigue (*β* = −0.78, *p* = 0.005). Fatigue had a direct effect on medication errors (*β* = 0.32, *p* = 0.003), while communication showed both direct and indirect effects on medication errors. The model explained 64.3% of the variance in communication, 45.4% in fatigue, and 29.8% in medication errors.

**Conclusion:**

A supportive nursing work environment facilitates effective interprofessional communication, which in turn mitigates fatigue and reduces medication errors. Communication functions as a key mediating mechanism within this structural pathway.

**Implications for Nursing Management:**

To reduce medication errors, nurse managers should prioritize fostering positive work environments, implementing structured communication protocols, and establishing systematic fatigue management strategies. Such organizational initiatives may enhance communication efficiency and strengthen patient safety outcomes.

## 1. Introduction

The World Health Organization (WHO) continues to emphasize patient safety as a global health priority and recently released a report underscoring the need for national frameworks to prevent medication errors and strengthen safety systems [1]. In South Korea, the Patient Safety Act was implemented in 2016, after which the number of reported patient safety incidents has shown a continuous upward trend. Notably, medication errors constitute the second most frequently reported category of patient safety incidents [2]. These errors not only pose direct threats to patient health and life but also impose a substantial global economic burden, with an estimated annual cost of approximately $42 billion [3]. The most recent WHO report further highlighted the urgency of preventive strategies given the widespread occurrence and serious consequences of medication‐related harm [1].

More than 50% of medication errors reportedly involve nurses, who serve as the final executors in the medication administration process [4]. Accordingly, identifying the causes of medication errors among nurses requires examination of a broad range of individual, environmental, and organizational factors. Previous studies have applied the theory of planned behavior to investigate individual‐level psychological determinants, such as attitudes and intentions [5], whereas other research has focused on environmental conditions, including workload, staffing levels, lighting, noise, and interruptions [6]. A recent systematic review reported that nurses perceive high workload, understaffing, fatigue, and environmental distractions as major contributors to medication administration errors [6]. However, medication errors are more accurately conceptualized as outcomes of complex and interrelated factors spanning organizational systems, environmental conditions, and interpersonal communication processes [7].

Ecological Systems Theory (EST), originally proposed by Bronfenbrenner [8], offers a comprehensive framework for understanding interactions between individuals and their environments and provides a useful lens for examining the multifactorial causes of medication errors among nurses. Among these factors, nurse fatigue has been consistently identified as a major risk factor. Shift work and excessive workload are known to impair concentration and cognitive performance, thereby increasing the likelihood of errors [9, 10]. Both the WHO and the National Coordinating Council for Medication Error Reporting and Prevention (NCCMERP) have identified fatigue as a primary contributor to medication errors [3, 11].

In addition, ineffective communication among healthcare professionals remains a critical contributor to medication errors, with approximately 60% of patient safety incidents attributed to communication failures [11, 12]. Recent studies have further demonstrated that supportive nursing work environments characterized by adequate staffing, effective leadership, and institutional support are associated with fewer medication errors and improved patient safety outcomes [13, 14].

To extend the theoretical foundation of the present study, Bronfenbrenner’s EST was applied to conceptualize how multilevel contextual factors shape medication‐related outcomes among nurses. EST posits that human behavior emerges from dynamic interactions across multiple environmental systems [8, 15]. In this study, the nursing work environment is conceptualized as the mesosystem, reflecting organizational resources, staffing adequacy, leadership, and institutional culture that influence nurses’ practice conditions [16, 17]. Communication among healthcare professionals is situated within the microsystem, representing immediate interpersonal processes embedded in daily nurse–physician interactions [18, 19]. Nurse fatigue is positioned within the individual system, reflecting physiological and psychological responses to workplace demands shaped by both micro‐ and mesosystem influences [20, 21]. Medication errors are conceptualized as behavioral outcomes arising from these multilevel system interactions, consistent with integrated patient safety frameworks [3, 7]. This ecological perspective provides a coherent theoretical rationale for the hypothesized pathways linking the nursing work environment, communication, fatigue, and medication errors.

**FIGURE 1:**
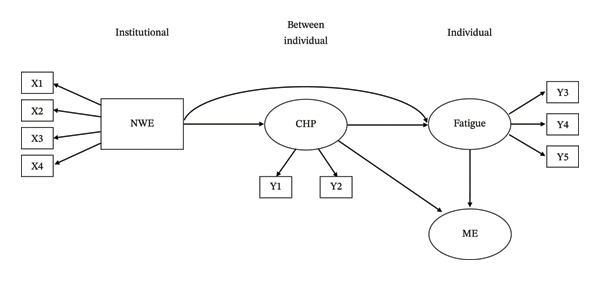
Hypothetical model. CHP = communication with health professionals; ME = medication error; NWE = nursing work environment; X1 = nurse participation in hospital affairs; X2 = nursing foundations for quality of care; X3 = nurse manager ability, leadership, and support for nurses; X4 = staffing and resource adequacy; Y1 = nurse–physician communication; Y2 = nurse–nurse communication; Y3 = depletive fatigue; Y4 = nervous fatigue; Y5 = chronic fatigue.

To explicitly articulate these theoretical linkages, the present study developed a structured set of hypotheses grounded in sequential ecological pathways. Specifically, a positive nursing work environment at the mesosystem level is hypothesized to enhance communication among healthcare professionals at the microsystem level, which in turn reduces fatigue at the individual level. Fatigue is expected to increase the likelihood of medication errors, whereas effective communication is anticipated to mitigate such risks. These hypotheses reflect both the core assumptions of EST and prior empirical evidence supporting cross‐level relationships among organizational, interpersonal, and individual factors. The hypothetical model proposed in this study is presented in Figure 1, and the study was conducted based on the following hypotheses:

 H1. The nursing work environment has a significant effect on communication with health professionals.
 H2. The nursing work environment has a significant effect on fatigue.
 H3. The nursing work environment has a significant indirect effect on fatigue mediated by communication with health professionals.
 H4. Communication with health professionals has a significant effect on fatigue.
 H5. Communication with health professionals mediates the relationship between the nursing work environment and medication errors.
 H6. Fatigue mediates the relationship between the nursing work environment and medication errors.
 H7. Communication with health professionals has a significant effect on medication errors.
 H8. Communication with health professionals has a significant indirect effect on medication errors via fatigue.
 H9. Fatigue has a significant effect on medication errors.

## 2. Methods

### 2.1. Study Design and Participants

This quantitative study employed convenience sampling to recruit clinical nurses responsible for medication administration in tertiary hospitals with more than 200 beds located in a metropolitan area of South Korea. Eligibility criteria included having a minimum of 1 year of clinical experience and active involvement in direct medication administration. Nurses working in outpatient departments, charge nurses, and nurses with less than 1 year of clinical experience were excluded to minimize potential confounding related to limited clinical exposure and role‐related differences.

Sample size determination followed established recommendations for structural equation modeling (SEM), which suggest a minimum of 200–400 participants or at least 10 to 15 participants per observed variable [22]. A power analysis based on maximum likelihood estimation further indicated that a sample size of 200 or more would provide adequate statistical power for model testing [23]. Beyond these general guidelines, sample adequacy was evaluated in accordance with methodological recommendations specific to maximum likelihood estimation in SEM. Previous studies have demonstrated that models of comparable complexity achieve stable parameter estimates with sample sizes exceeding 200 [24, 25]. Additionally, the application of the SEM power analysis framework proposed by Moshagen and Erdfelder [23] indicates that a minimum sample size of 200 yields a model power of 0.99 for detecting structural relationships of moderate magnitude. Accordingly, the final sample of 230 nurses exceeded all recommended thresholds and was deemed statistically sufficient to ensure reliable parameter estimation and robust model evaluation. Of the 240 self‐administered questionnaires distributed, 230 complete responses were returned and included in the final analysis.

### 2.2. Instruments

Sociodemographic and work‐related characteristics were collected using structured questionnaires and included age, sex, marital status, education level, monthly income, total clinical experience, clinical experience in the current unit, work area (e.g., medical ward or intensive care unit), and work shift type (e.g., three‐shift system). In addition, lifestyle‐related variables, including alcohol consumption and exercise habits, were assessed.

The nursing work environment was assessed using the Korean version of the Practice Environment Scale of the Nursing Work Index (K‐PES‐NWI), originally developed by Lake [26] and revised by Cho et al. [27]. This 26‐item instrument comprises four subscales: nurse participation in hospital affairs (9 items), nursing foundations for quality of care (9 items), nurse manager ability, leadership, and support for nurses (4 items), and staffing and resource adequacy (4 items). Items are rated on a 4‐point Likert scale ranging from 1 (“strongly disagree”) to 4 (“strongly agree”), with higher scores indicating more positive perceptions of the nursing work environment. Cronbach’s *α* was 0.93 at the time of the scale revision [27] and 0.92 in the present study.

Communication with health professionals was measured using the Korean version of the Nurse–Physician Communication Scale, originally developed by Shortell et al. [28] and adapted by Cho et al. [18]. This 27‐item instrument consists of two subscales: nurse–physician communication (16 items) and nurse–nurse communication (11 items). Each item is rated on a 5‐point Likert scale ranging from 1 (“strongly disagree”) to 5 (“strongly agree”), with higher scores reflecting more effective communication. Cronbach’s *α* was 0.89 in both the original validation study [18] and the present study.

Fatigue was assessed using the Nurse Fatigue Scale developed by Jang [29]. This 24‐item scale comprises three subscales assessing depletive, nervous, and chronic fatigue. Items are rated on a 5‐point Likert scale, yielding total scores ranging from 24 to 120, with higher scores indicating greater levels of fatigue. Cronbach’s *α* was 0.93 at the time of scale development and 0.94 in the present study.

Medication errors were measured using the Medication Error Reporting Scale developed by Lee [30] and revised by Kim et al. [31]. Of the original 18 items, eight items with item–total correlation coefficients below 0.30 (Items 1, 3, 4, 5, 8, 9, 12, and 16) were excluded, resulting in a 10‐item scale. Each item assessed the frequency of medication errors occurring within the previous 3 months using a 6‐point Likert scale ranging from 0 (none) to 5 (five or more times), with higher scores indicating more frequent medication errors. Cronbach’s *α* was 0.52 in Kim et al. [32] and 0.60 in the present study.

### 2.3. Data Collection and Ethical Considerations

This study was conducted between February 23 and March 30, 2023, following approval from the Institutional Review Board (IRB) of Kyungpook University Hospital (IRB No.: 2023‐01‐015‐001). During the data collection period, the researcher visited the nursing departments of four tertiary hospitals, explained the purpose of the study to hospital administrators, and obtained their cooperation and permission. Participants were recruited through announcements posted in easily accessible locations at nurses’ stations. Nurses who expressed interest in participating received detailed information both verbally and in written form regarding the study’s purpose and procedures, voluntary participation and anonymity, potential benefits and risks, and their right to withdraw from the survey at any time without penalty.

Nurses who voluntarily agreed to participate provided written informed consent and subsequently completed the questionnaire. Completed questionnaires were sealed in individual envelopes and collected directly by the researcher. As a token of appreciation, participants received a small gift upon survey completion. All collected data will be securely stored in a locked personal file cabinet in accordance with IRB regulations for a period of 3 years and will be permanently destroyed following the data retention period.

### 2.4. Data Analyses

Data were analyzed using IBM SPSS version 22.0 and AMOS version 22.0. Participants’ general characteristics were summarized using descriptive statistics, including frequencies, percentages, means, and standard deviations. The internal consistency reliability of each measurement scale was evaluated using Cronbach’s *α*.

Prior to conducting SEM, data normality was assessed by examining means, standard deviations, skewness, and kurtosis. Acceptable thresholds for normality were defined as |skewness| < 3 and |kurtosis| < 10 [22]. Pearson’s correlation coefficients were calculated to examine relationships among the study variables. Multicollinearity was assessed using tolerance and variance inflation factor (VIF) values, with correlation coefficients below 0.80 and VIF values below 10 indicating the absence of multicollinearity concerns [33].

SEM parameter estimates were obtained using maximum likelihood estimation. Confirmatory factor analysis (CFA) was performed to evaluate the relationships between latent constructs and their observed indicators. Convergent validity was assessed using standardized regression weights (SRW ≥ 0.5), construct reliability (CR ≥ 0.7), and average variance extracted (AVE ≥ 0.5) [33]. Discriminant validity was evaluated by comparing AVE values with the squared interconstruct correlations, with discriminant validity considered adequate when AVE exceeded the corresponding squared correlations [34].

Model fit was evaluated using multiple absolute and incremental fit indices, including *χ* ^2^, normed *χ* ^2^ (CMIN/DF), RMR, SRMR, RMSEA, GFI, AGFI, TLI, and CFI. The statistical significance of parameter estimates was determined using SRW, construct reliability (CR), and *p* values. The explanatory power of exogenous variables was examined using squared multiple correlations (SMC). Indirect and total effects were tested using bootstrapping procedures, and phantom variables were employed to assess specific indirect effects.

## 3. Results

### 3.1. Sociodemographic and Work‐Related Characteristics

Table 1 summarizes the participants’ general characteristics. A total of 230 nurses were included in the analysis, of whom 92.2% were female. The mean age of participants was 29.25 years (SD = 5.07), with the majority (57.8%) aged 26–30 years. Most participants had graduated from university or college (89.1%) and were unmarried (76.1%). Regarding monthly income, 55.2% reported earnings of 250–300 (10,000 won). The mean total clinical experience was 6.33 years (SD = 5.00), with 33.5% of participants reporting 4–6 years of experience. The mean duration of clinical experience in the current work area was 2.74 years (SD = 1.68), with 43.5% reporting 2–3 years.

**TABLE 1:** General participant characteristics (*N* = 230).

Characteristics	Category	*n*	(%)	Mean ± SD	Range
Gender	Man	18	(7.8)		
	Woman	212	(92.2)		

Age (years)	< 26	42	(18.3)	29.25 ± 5.07	23–53
	26–30	133	(57.8)		
	31–40	45	(19.6)		
	> 40	10	(4.3)		

Education	College	7	(3.0)		
	University	205	(89.1)		
	Graduate School	18	(7.8)		

Marriage	No	175	(76.1)		
	Yes	55	(23.9)		

Monthly income (10,000 won)	< 250	34	(14.8)		
	250–300	127	(55.2)		
	301–350	42	(18.3)		
	> 350	27	(11.7)		

Clinical experience (years)	1–3	71	(30.9)	6.33 ± 5.00	1–30
	4–6	77	(33.5)		
	7–15	67	(29.1)		
	> 15	15	(6.5)		

Clinical experience in current area (years)	1	70	(30.4)	2.74 ± 1.68	1–10
	2–3	100	(43.5)		
	> 3	60	(26.1)		

Work area	Medical ward	41	(17.8)		
	Medical ICU	53	(23.0)		
	Surgical ward	44	(19.1)		
	Surgical ICU	48	(20.9)		
	Emergency room	44	(19.1)		

Work type	Three‐shift	224	(97.4)		
	Two‐shift	3	(1.3)		
	First shift	3	(1.3)		

Alcohol drinking	Yes	154	(67.0)		
	No	76	(33.0)		

Exercise	Regular (3 times/week)	68	(29.6)		
	None	65	(28.3)		
	Irregular	97	(42.2)		

Abbreviation: ICU = intensive care unit.

Participants most frequently worked in medical intensive care units (23.0%), followed by surgical intensive care units (20.9%), surgical wards (19.1%), emergency rooms (19.1%), and medical wards (17.8%). The vast majority worked under a three‐shift system (97.4%). With respect to lifestyle factors, 67.0% reported alcohol consumption, and 42.2% reported engaging in irregular exercise.

### 3.2. Descriptive Statistics and Normality Testing of Measured Variables

Table 2 presents the descriptive statistics and normality test results for all measured variables. The mean score for the nursing work environment was 63.47 (SD = 9.90). Mean scores for the subscales were as follows: nurse participation in hospital affairs, 20.80 (SD = 3.97); nursing foundations for quality of care, 23.83 (SD = 3.36); nurse manager ability, leadership, and support for nurses, 10.42 (SD = 2.02); and staffing and resource adequacy, 8.41 (SD = 2.23). The mean score for communication with health professionals was 97.46 (SD = 11.80). Mean subscale scores were 42.27 (SD = 9.11) for nurse–physician communication and 37.19 (SD = 5.63) for nurse–nurse communication. The mean fatigue score was 75.87 (SD = 15.62), with subscale means of 50.38 (SD = 11.31) for depletive fatigue, 10.61 (SD = 2.18) for nervous fatigue, and 14.89 (SD = 3.62) for chronic fatigue. The mean medication error score was 6.43 (SD = 5.47). Assessment of normality indicated that skewness values ranged from 0.01 to 0.96 and kurtosis values ranged from 0.08 to 1.88, all of which were within acceptable thresholds.

**TABLE 2:** Descriptive statistics of the measured variables.

	Mean	SD	Actual range	Possible range	Skewness	Kurtosis
NWE	63.47	9.90	33.0–104.0	26.0–104.0	−0.01	1.88
Nurse participation in hospital affairs	20.80	3.97	9.0–36.0	9.0–36.0	0.01	1.54
Nursing foundations for quality of care	23.83	3.36	12.0–36.0	9.0–36.0	−0.26	1.52
Nurse manager ability, leadership, and support for nurses	10.42	2.02	4.0–16.0	4.0–16.0	−0.34	0.60
Staffing and resource adequacy	8.41	2.23	4.0–16.0	4.0–16.0	0.33	0.08
CHP	79.46	11.80	48.0–107.0	27.0–135.0	−0.12	−0.41
Nurse–physician communication	42.27	9.11	18.0–69.0	16.0–80.0	−0.01	−0.24
Nurse–nurse communication	37.19	5.63	23.0–49.0	11.0–55.0	−0.44	−0.45
Fatigue	75.87	15.62	35.0–117.0	24.0–120.0	−0.15	−0.62
Depletive	50.38	11.31	22.0–78.0	16.0–80.0	−0.21	−0.50
Nervous	10.61	2.18	5.0–15.0	3.0–15.0	−0.31	−0.27
Chronic	14.89	3.62	7.0–24.0	5.0–25.0	−0.03	−0.87
Medication error	6.43	5.47	0.0–27.0	0.0–40.0	0.96	0.86

*Note:* CHP = communication with health professionals.

Abbreviation: NWE = nursing work environment.

### 3.3. Correlations Among Measured Variables

Table 3 presents the correlation matrix for the measured variables included in the hypothetical model. Tolerance values ranged from 0.30 to 0.68, and VIF values ranged from 1.47 to 3.37, indicating no evidence of multicollinearity. Pearson correlation coefficients ranged from −0.20 to 0.79, further supporting the absence of multicollinearity among the study variables.

**TABLE 3:** Correlations between measurement variables.

	X1	X2	X3	X4	Y1	Y2	Y3	Y4	Y5	Tolerance	VIF
X1	1									0.30	3.32
X2	0.79^∗∗^	1								0.30	3.37
X3	0.60^∗∗^	0.58^∗∗^	1							0.55	1.82
X4	0.56^∗∗^	0.58^∗∗^	0.49^∗∗^	1						0.53	1.89
Y1	0.47^∗∗^	0.39^∗∗^	0.29^∗∗^	0.46^∗∗^	1					0.67	1.50
Y2	0.37^∗∗^	0.49^∗∗^	0.42^∗∗^	0.32^∗∗^	0.24^∗∗^	1				0.68	1.47
Y3	−0.36^∗∗^	−0.37^∗∗^	−0.40^∗∗^	−0.45^∗∗^	−0.39^∗∗^	−0.38^∗∗^	1			0.33	3.05
Y4	−0.24^∗∗^	−0.21^∗∗^	−0.24^∗∗^	−0.20^∗∗^	−0.32^∗∗^	−0.23^∗∗^	0.58^∗∗^	1		0.64	1.56
Y5	−0.35^∗∗^	−0.34^∗∗^	−0.35^∗∗^	−0.34^∗∗^	−0.35^∗∗^	−0.39^∗∗^	0.76^∗∗^	0.49^∗∗^	1	0.40	2.48

*Note:* X1 =nurse participation in hospital affairs; X2 = nursing foundations for quality of care; X3 = nurse manager ability, leadership, and support for nurses; X4 = staffing and resource adequacy; Y1 = nurse–physician communication; Y2 = nurse–nurse communication; Y3 = depletive fatigue; Y4 = nervous fatigue; Y5 = chronic fatigue.

Abbreviation: VIF = variance inflation factor.

^∗^ *p* < 0.05.

^∗∗^ *p* < 0.001.

### 3.4. Measurement Model Verification: CFA and Validity

CFA was conducted to evaluate construct validity by examining factor loadings and overall model fit [33]. Multiple fit indices were used to assess goodness of fit, as reliance on a single index is not recommended [22].

The CFA results indicated that the absolute fit index, *χ* ^2^ = 82.75 (*p* < 0.001), did not meet the recommended threshold. However, the relative fit index demonstrated acceptable fit (*χ* ^2^/df = 2.67). Other fit indices further supported adequate model fit, including RMSEA = 0.08, SRMR = 0.05, GFI = 0.94, AGFI = 0.89, TLI = 0.93, and CFI = 0.95 (Table 4).

**TABLE 4:** Measurement model fit indices.

Fit index	*χ* ^2^ (*p*)	*χ* ^2^/df	RMSEA	SRMR	GFI	AGFI	TLI	CFI
Hypothetical Model	*p* < 0.001	2.67 (82.75/31)	0.08	0.05	0.94	0.89	0.93	0.95
Reference	*p* > 0.05	≤ 3	≤ 0.08	≤ 0.08	≥ 0.90	≥ 0.85	≥ 0.90	≥ 0.90

*Note:* AGFI, adjusted goodness‐of‐fit index; GFI, goodness‐of‐fit index; RMSEA, root mean square error of approximation; SRMR, standardized root mean square residual.

Abbreviations: CFI, comparative fit index; TLI, Tucker–Lewis index.

Convergent validity was assessed using standardized factor loadings, AVE, and construct reliability (CR) [22]. As shown in Table 5, factor loadings for the nursing work environment ranged from 0.67 to 0.89, with an AVE of 0.87 and CR of 0.96, satisfying all recommended criteria. The fatigue construct demonstrated factor loadings ranging from 0.61 to 0.82, with an AVE of 0.77 and a CR of 0.91. Communication with health professionals showed a factor loading of 0.51, an AVE of 0.52, and a CR of 0.70, indicating acceptable convergent validity.

**TABLE 5:** Standardized estimates for the measurement model.

Factor	*B*	*β*	S.E.	C.R.	*p* value	AVE	CR
NWE							
X1	1.00	0.88				0.87	0.96
X2	0.86	0.89	0.05	17.17	< 0.001		
X3	0.88	0.68	0.08	11.63	< 0.001		
X4	0.96	0.67	0.08	11.36	< 0.001		
CHP							
Y1	1.00	0.51				0.52	0.70
Y2	0.90	0.51	0.14	6.45	< 0.001		
Fatigue							
Y3	1.46	0.92	0.15	9.79	< 0.001	0.77	0.91
Y4	1.00	0.61					
Y5	1.33	0.82	0.14	9.65	< 0.001		

*Note:* CHP, communication with health professionals; X1 = nurse participation in hospital affairs; X2 = nursing foundations for quality of care; X3 = nurse manager ability, leadership, and support for nurses; X4 = staffing and resource adequacy; Y1 = nurse–physician communication; Y2 = nurse–nurse communication; Y3 = depletive fatigue; Y4 = nervous fatigue; Y5 = chronic fatigue.

Abbreviations: AVE, average variance extracted; CR, construct reliability; C.R., critical ratio; NWE, nursing work environment; S.E., standard error.

Discriminant validity was evaluated by comparing AVE values with the squared interconstruct correlations [22]. As presented in Table 6, the AVE values for the nursing work environment (0.87) and communication with health professionals (0.52) exceeded the squared correlation between these constructs (0.36), supporting discriminant validity. All remaining constructs also satisfied this criterion.

**TABLE 6:** Correlation coefficients among latent variables and squared correlation coefficients.

	AVE	NWE	CHP	Fatigue
NWE	0.87	1	0.36^∗^	0.21^∗^
CHP	0.52	0.60	1	0.25^∗^
Fatigue	0.77	−0.46	−0.50	1

*Note:* CHP = communication with health professionals.

Abbreviation: NWE = nursing work environment.

^∗^Square of correlation coefficient.

### 3.5. Structural Model Evaluation

The structural model was evaluated to test the hypothesized relationships among the study variables. The model demonstrated acceptable fit, with *χ* ^2^/df = 2.98, RMSEA = 0.08, SRMR = 0.06, GFI = 0.91, AGFI = 0.86, TLI = 0.90, and CFI = 0.93 (Table 7).

**TABLE 7:** Hypothetical model fit indices.

Fit index	*χ* ^2^ (*p*)	*χ* ^2^/df	RMSEA	SRMR	GFI	AGFI	TLI	CFI
Hypothetical Model	*p* < 0.001	2.98 (119.26/40)	0.08	0.06	0.91	0.86	0.90	0.93
Reference	*p* > 0.05	≤ 3	≤ 0.08	≤ 0.08	≥ 0.90	≥ 0.85	≥ 0.90	≥ 0.90

*Note:* AGFI = adjusted goodness‐of‐fit index; GFI = goodness‐of‐fit index; RMSEA = root mean square error of approximation; SRMR = standardized root mean square residual.

Abbreviations: CFI, comparative fit index; TLI, Tucker–Lewis index.

**FIGURE 2:**
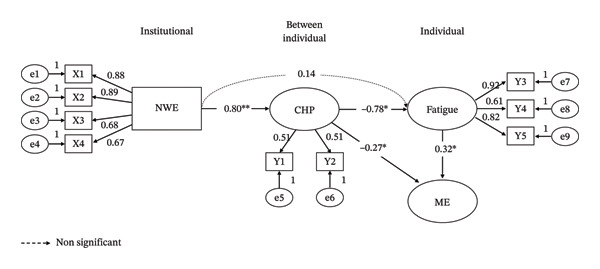
Structural equation model of medication errors in clinical nursing. ^∗^ *p* < 0.05, ^∗∗^ *p* < 0.001. Solid arrows indicate statistically significant paths, whereas dashed arrows represent nonsignificant paths. CHP = communication with health professionals; ME = medication error; NWE = nursing work environment; X1 = nurse participation in hospital affairs; X2 = nursing foundations for quality of care; X3 = nurse manager ability, leadership, and support for nurses; X4 = staffing and resource adequacy; Y1 = nurse–physician communication; Y2 = nurse–nurse communication; Y3 = depletive fatigue; Y4 = nervous fatigue; Y5 = chronic fatigue.

Path analysis results are presented in Table 8. The nursing work environment had a significant positive effect on communication with health professionals (*β* = 0.80, *p* < 0.001), explaining 64.3% of the variance in communication. Communication with health professionals had a significant negative effect on fatigue (*β* = −0.78, *p* = 0.005), whereas the direct effect of the nursing work environment on fatigue was not statistically significant (*β* = 0.14, *p* = 0.535). Together, these variables accounted for 45.4% of the variance in fatigue. Medication errors were significantly predicted by communication with health professionals (*β* = −0.27, *p* = 0.010) and fatigue (*β* = 0.32, *p* = 0.003), collectively explaining 29.8% of the variance in medication errors. The final SEM with standardized path coefficients is presented in Figure 2.

**TABLE 8:** Estimation of path coefficients in the hypothetical model.

Endogenous variables	Exogenous variables	*B*	Β	S.E.	C.R.	*p*	SMC
CHP	NWE	0.71	0.80	0.09	7.92	< 0.001	0.643

Fatigue	NWE	0.16	0.14	0.26	0.62	0.535	0.454
	CHP	−1.01	−0.78	0.36	−2.81	0.005	

ME	CHP	−0.54	−0.27	0.21	−2.59	0.010	0.298
	Fatigue	0.50	0.32	0.15	3.25	0.003	

*Note:* CHP, communication with health professionals.

Abbreviations: C.R., critical ratio; ME, medication error; NWE, nursing work environment; S.E., standard error; SMC, squared multiple correlations.

### 3.6. Direct, Indirect, and Total Effects

Table 9 summarizes the results of the analyses examining direct, indirect, specific indirect, and total effects. The nursing work environment exerted significant direct and total effects on communication with health professionals (*β* = 0.80, *p* = 0.001). Although the direct effect of the nursing work environment on fatigue was not statistically significant (*β* = 0.14, *p* = 0.583), both the specific indirect effect mediated through communication with health professionals (*β* = −0.63, *p* = 0.001) and the total effect (*β* = −0.49, *p* = 0.001) were significant.

**TABLE 9:** Standardized estimates of direct, indirect, and total effects in the hypothetical model.

Exogenous variables	Endogenous variables	*B*	*β*	S.E.	*p*	Direct effect (*p*)	Indirect effect (*p*)	Total effect (*p*)	SMC
NWE	⟶ CHP	0.71	0.80	0.09	< 0.001	0.80 (0.001)		0.80 (0.001)	0.643

NWE	⟶ Fatigue	0.16	0.14	0.26	0.535	0.14 (0.583)		−0.49 (0.001)	0.454
NWE ⟶ CHP	⟶ Fatigue	−0.72	−0.63	0.84	0.001		−0.63 (0.001)		
CHP	⟶ Fatigue	−1.01	−0.78	0.36	0.005	−0.78 (0.001)		−0.78 (0.001)	

NWE ⟶ CHP	⟶ ME	−0.39	−0.22	0.17	0.041		−0.22 (0.041)	−0.38 (0.001)	0.298
NWE ⟶ Fatigue	⟶ ME	0.08	0.05	0.65	0.583		0.05 (0.583)		
CHP	⟶ ME	−0.54	−0.27	0.21	0.010	−0.27 (0.041)		−0.53 (0.001)	
CHP ⟶ Fatigue	⟶ ME	−0.50	−0.25	0.87	0.003		−0.25 (0.003)		
Fatigue	⟶ ME	0.50	0.32	0.15	0.001	0.32 (0.003)		0.32 (0.003)	

*Note:* CHP = communication with health professionals.

Abbreviation: NEW = nursing work environment.

Communication with health professionals demonstrated significant direct and total effects on fatigue (*β* = −0.78, *p* = 0.001). The nursing work environment also showed a significant specific indirect effect on medication errors through communication with health professionals (*β* = −0.22, *p* = 0.041). In contrast, the indirect effect mediated by fatigue was not significant (*β* = 0.05, *p* = 0.583); however, the total effect of the nursing work environment on medication errors remained significant (*β* = −0.38, *p* = 0.001).

Communication with health professionals exhibited both a significant direct effect on medication errors (*β* = −0.27, *p* = 0.041) and a significant specific indirect effect mediated by fatigue (*β* = −0.25, *p* = 0.003), resulting in a significant total effect (*β* = −0.53, *p* = 0.001).

Finally, fatigue had a significant direct and total effect on medication errors (*β* = 0.32, *p* = 0.003).

### 3.7. Hypothesis Testing

With respect to hypothesis testing, Hypothesis 1 was supported, as the nursing work environment had a significant effect on communication with health professionals (*β* = 0.80, *p* = 0.001). Hypothesis 2 was rejected because the direct effect of the nursing work environment on fatigue was not statistically significant (*β* = 0.14, *p* = 0.583). Hypothesis 3 was supported, indicating a significant indirect effect of the nursing work environment on fatigue mediated by communication with health professionals (*β* = −0.63, *p* = 0.001). Hypothesis 4 was also supported, demonstrating a significant effect of communication with health professionals on fatigue (*β* = −0.78, *p* = 0.001). Hypothesis 5 was supported, confirming that communication with health professionals mediated the relationship between the nursing work environment and medication errors (*β* = −0.22, *p* = 0.041). Hypothesis 6 was rejected, as no significant mediating effect of fatigue was observed (*β* = 0.05, *p* = 0.583). Hypothesis 7 was supported, with communication with health professionals exerting a significant direct effect on medication errors (*β* = −0.27, *p* = 0.041). Hypothesis 8 was also supported, indicating a significant indirect effect of communication with health professionals on medication errors via fatigue (*β* = −0.25, *p* = 0.003). Finally, Hypothesis 9 was supported, as fatigue had a significant effect on medication errors (*β* = 0.32, *p* = 0.003).

Overall, seven hypotheses (H1, H3, H4, H5, H7, H8, and H9) were supported, whereas two hypotheses (H2 and H6) were not supported.

## 4. Discussion

This study examined the structural relationships among the nursing work environment, communication among healthcare professionals, fatigue, and medication errors, grounded in Bronfenbrenner’s [8] EST. The findings demonstrate that a supportive nursing work environment significantly enhances interprofessional communication. This result is consistent with international evidence indicating that positive organizational culture and effective leadership are central drivers of collaborative communication in healthcare settings [16, 17]. For example, Aiken et al. [16] reported that hospitals with favorable work environments achieved higher levels of nurse–physician collaboration and superior patient safety outcomes across 12 countries. Similarly, Lake et al. [17] emphasized that poor work environments undermine teamwork and contribute to communication failures. Further supporting this perspective, Vermeir et al. [19], in a narrative review of healthcare communication, concluded that ineffective communication among healthcare professionals is a major contributor to medical errors, delayed treatment, and diminished quality of care. Collectively, these findings underscore the critical importance of structured and collaborative communication systems within healthcare organizations. In this context, the present results suggest that strengthening staffing adequacy and leadership support, which are key components of the nursing work environment, together with implementing structured communication strategies such as Situation–Background–Assessment–Recommendation (SBAR) and interprofessional training programs, may effectively promote teamwork and reduce medication errors.

In interpreting these findings, it is important to acknowledge that the construct “communication with health professionals” demonstrated psychometric indices at the lower bound of commonly accepted thresholds. Nevertheless, this construct was retained because of its theoretical centrality within the EST framework, its strong internal consistency (Cronbach’s *α* = 0.89), and established psychometric guidelines indicating that factor loadings of ≥ 0.50 and AVE values near 0.50 may be considered acceptable when supported by theoretical justification and adequate composite reliability [22, 33, 34]. Importantly, this construct exhibited significant structural effects, including a key mediating pathway linking mesosystem‐level and individual‐level factors. Retaining this latent variable therefore preserved both the conceptual coherence of the theoretical model and its empirical explanatory strength.

The mediating role of interprofessional communication in reducing fatigue, as well as its indirect effect on medication errors, was also supported. These findings align with prior research demonstrating that effective communication among healthcare professionals can reduce work‐related stress and psychological burden, thereby mitigating fatigue [20, 35]. For instance, Khamisa et al. [35] found that team‐based communication and organizational support were critical factors in reducing nurse fatigue and burnout in South Africa. Similarly, Geiger‐Brown et al. [36] reported that fatigue among shift‐working nurses impairs cognitive function and increases the likelihood of clinical errors. More recent studies have further highlighted that extended working hours, inadequate rest, and poorly managed shift schedules substantially contribute to nurse fatigue and elevate the risk of adverse patient outcomes, including medication errors [21, 37]. Taken together, these findings suggest that interventions such as optimizing shift schedules, incorporating structured debriefing practices, and promoting standardized communication tools like SBAR may be effective strategies for reducing nurse fatigue and enhancing patient safety.

This study further confirmed the indirect effect of the nursing work environment on medication errors through communication, underscoring that the work environment extends beyond physical infrastructure to encompass organizational culture and interpersonal dynamics [16, 38]. Cho et al. [38] reported that collaborative nurse–physician relationships are associated with fewer patient safety incidents, including medication errors. In addition, Tariq et al. [39] identified communication breakdowns and excessive workloads as major contributors to medication errors and emphasized the importance of implementing standardized communication protocols and adopting electronic health records (EHRs). In this context, equipping nurses with mobile health technologies or EHR‐linked devices may further facilitate real‐time communication and contribute to reductions in medication errors.

The direct effect of fatigue on medication errors observed in the present study is consistent with recent empirical evidence. Bell et al. [40] demonstrated that nurse fatigue, particularly when associated with night shifts and extended working hours, is significantly related to an increased risk of medication errors and near misses, primarily through impaired cognitive performance and attentional lapses. Similarly, Farag et al. [41] found that even moderate levels of self‐reported fatigue significantly increase the likelihood of medication administration errors. Collectively, these findings highlight the need for organizational interventions, including optimized shift scheduling, scheduled rest periods, and evidence‐based fatigue management strategies, to enhance clinical performance and patient safety.

Moreover, the Joint Commission [42] reported that nearly 70% of sentinel events were attributable to communication failures. Haig et al. [43] demonstrated that structured communication tools, such as Situation–Background–Assessment–Recommendation (SBAR), can effectively reduce the occurrence of such events. In light of these findings, the present results indicate that the nursing work environment, communication among healthcare professionals, and fatigue represent interrelated factors that jointly influence medication errors. Accordingly, a comprehensive, system‐level approach that integrates improvements in the work environment, communication skills training, and fatigue management programs is essential for meaningful reductions in medication errors.

Future research should further explore the role of digital platforms, including EHR‐integrated communication tools and artificial intelligence–driven monitoring systems, in enhancing real‐time information exchange and medication safety. Such approaches are consistent with global strategies aimed at advancing patient safety initiatives [44].

An additional consideration pertains to measurement issues related to the medication error scale that necessitated the removal of several items. Importantly, the low item–total correlations were attributable to systematic factors rather than random measurement error. Several excluded items captured behaviors that occur infrequently in tertiary hospital settings, such as improper dilution or dosing, resulting in highly skewed distributions and limited variance. Other items primarily reflected documentation‐related lapses, including delayed charting or failure to record adverse reactions, rather than actual medication administration behaviors, indicating insufficient conceptual alignment with the intended construct. Furthermore, some items were highly dependent on institution‐specific workflow policies, such as verification of patient self‐administration or timing deviations exceeding 1 hour, which likely contributed to inconsistent interpretation across hospitals.

Consistent with these observations, the same items were previously identified as psychometrically weak in the revised validation study by Kim et al. [31], further supporting their exclusion in the present analysis. Consequently, removing these items resulted in a more theoretically coherent and psychometrically robust 10‐item medication error scale.

### 4.1. Study Limitations

This study has several limitations that warrant consideration. First, the instrument used to assess medication errors demonstrated limited internal consistency and did not account for the severity or clinical consequences of individual errors. This limitation may have constrained the precision of outcome interpretation. Future research should focus on the development and validation of more refined measurement tools that incorporate error severity, clinical impact, and weighted scoring approaches. Second, although Bronfenbrenner’s EST provided the guiding framework, the analysis was restricted to the individual, interpersonal (microsystem), and institutional (mesosystem) levels. Broader contextual influences, including organizational policy, national health system structures, and cultural factors, were not examined, thereby narrowing the overall ecological scope. Third, the instrument used to assess the nursing work environment did not capture physical environmental conditions or staffing adequacy, both of which may influence the occurrence of medication errors. This omission limited the ability to evaluate workplace conditions in a more comprehensive manner. Finally, the sample comprised nurses from four tertiary hospitals within a single metropolitan area, which may limit the generalizability of the findings to other geographic regions or hospital settings. Moreover, data collection took place during the COVID‐19 pandemic, which restricted researcher access to clinical sites and reduced the pool of eligible participants. Consequently, although the sample size was statistically sufficient for SEM, the representativeness of the sample may be limited. Future studies employing broader sampling strategies across diverse hospitals and regions are recommended.

Despite these limitations, the identified structural relationships are theoretically grounded in EST and supported by international literature. Therefore, the proposed model may be applicable to other hospital settings with similar organizational structures. However, caution is warranted when generalizing the results to different healthcare systems or cultural contexts.

## 5. Conclusions

Using SEM, this study examined the relationships among the nursing work environment, interprofessional communication, fatigue, and medication errors among clinical nurses, grounded in Bronfenbrenner’s EST. The proposed model, which specified the nursing work environment as an exogenous variable and interprofessional communication, fatigue, and medication errors as endogenous variables, demonstrated good overall fit. Based on these findings, strategies to reduce medication errors among clinical nurses should prioritize improvements in the nursing work environment, strengthen interprofessional communication, and implement targeted fatigue management programs supported by organizational policies.

## Author Contributions

Junekyu Kim and Yeoungsuk Song contributed conception and design. Junekyu Kim acquired the data. Junekyu Kim and Yeoungsuk Song wrote the draft and revised the manuscript.

## Funding

This research received no external funding.

## Disclosure

All authors approved the final manuscript and agree to be accountable for all aspects on accuracy or integrity of any part.

## Ethics Statement

This study has been approved by the Ethics Committee of Kyungpook National University (KNU IRB: 2023‐01‐015‐001).

## Conflicts of Interest

The authors declare no conflicts of interest.

## Data Availability

The data that support the findings of this study are available on request from the corresponding author. The data are not publicly available due to privacy or ethical restrictions.
